# Association between second mesiobuccal missed canals and apical periodontitis in maxillary molars of a Chilean subpopulation

**DOI:** 10.4317/jced.60156

**Published:** 2023-03-01

**Authors:** Fernando Peña-Bengoa, Carolina Cáceres, Sven-Eric Niklander, Patricio Meléndez

**Affiliations:** 1 Departament of Endodontics, Dentistry Faculty, Universidad Andres Bello, Viña del Mar, Chile; 2 Unit of Oral Pathology and Medicine, Dentistry Faculty, Universidad Andres Bello, Viña del Mar, Chile; 3 Department of Oral and Maxillofacial Imaging, Dentistry Faculty, Universidad Andres Bello, Viña del Mar, Chile

## Abstract

**Background:**

To determine the frequency of missed second mesiobuccal canals (MB2) and apical periodontitis in maxillary molars of a Chilean subpopulation using cone beam computed tomography (CBCT).

**Material and Methods:**

Two previously calibrated operators evaluated CBCTs with a total of 588 upper molars, of which 179 endodontically treated molars were selected. Axial tomographic slices were used to study the frequency and association between the presence of apical periodontitis and untreated MB2 canals.

**Results:**

Of the 179 endodontically treated molars, 45.78% (84) presented MB2 missed canals. Of the upper molars that presented MB2 missed canals, 70% were associated with apical periodontitis, which was statistically significant (*p*< 0.0001). Sixty-two corresponded to first molars (74%) and 22 to second molars (26%). Of the first molars, 34 (54.8%) presented with apical periodontitis and MB2 missed canals (*p*< 0.0001), while 12 (54.4%) of the second molars presented this association (*p* = 0.081).

**Conclusions:**

MB2 missed canals are associated with a high degree of apical periodontitis and may be an important predictor of endodontic prognosis of upper molars.

** Key words:**Endodontics, apical periodontitis, cone beam computed tomography, missed canals, maxillary molars.

## Introduction

Post-treatment apical periodontitis (AP) is an inflammatory lesion affecting the peri radicular tissues caused primarily by a secondary or persistent infection of the root canal system (RCS) (1). The available literature reports that between 17% and 23% of AP can be associated with untreated canals (2), with molars being the most affected teeth, followed by premolars, incisors, and canines (2-4). Post-treatment AP is frequently related to failures during endodontic treatment (5,6), where instrumentation, disinfection, and a three-dimensional obturation of the RCS are the key stages to achieve success (1,5-7).

One of the most frequent causes of post-treatment AP is an incomplete filling of the RCS (7,8). This is not only associated with incomplete obturation, but also with missed and untreated canals which have the potential to initiate the disease, especially in teeth with necrotic pulps (9). The persistence of organic tissue in unprepared areas of the RCS favors the proliferation of microorganisms, thus initiating apical periodontitis and contributing to endodontic treatment failure (10,11).

Upper molars are the most frequently teeth affected by apical periodontitis due to untreated canals (12,13), a cause attributed primarily to missing the second mesiobucal (MB2) canal. The reported prevalence of post-treatment AP associated with untreated MB2 canal is high, varying from 50–90% depending on the affected tooth (first or second molar), and the study methodology (10,14-16). A good knowledge of the tooth anatomy, associated with good endodontic planning and a proper use magnification, are essential tools to locate and treat the MB2 canal properly (17,18).

The endodontic planning is based on clinical and imaging data, where cone beam computed tomography (CBCT) is becoming more frequent for endodontic planning. The use of CBCT is considered a valuable tool for endodontic planning as increases the likelihood of achieving a successful treatment (9,19). It allows the visualization of the internal dental anatomy facilitating a more efficient and effective approach to the RCS. It also provides a precise three-dimensional representation eliminating the limitations of conventional radiographs (20).

The literature regarding the association between AP and untreated canals is diverse. The aim of this study was to analyze the frequency and association of AP with MB2 missed canals in upper molars in a cohort of patients from the Region of Valparaíso, Chile.

## Material and Methods

This retrospective cohort study received ethical approval from the Ethical and Scientific Committee of the Dentistry Faculty of Universidad Andrés Bello (resolution number 6821).

-Sample selection

The sample size was determined using the known population formula, applied to the population of the continental territory of the Region of Valparaíso, Chile, obtained from the national registry (21), using a 5% significance level. The expected prevalence of missed canals in upper molars was of 40% (2). The obtained sample size was of 147 endodontically treated upper molars.

-Imaging analysis

The imaging analysis was performed independently by two operators previously calibrated using the ICAT vision software (Imaging Sciences International, Hatfield, United States) in a dark room with regulated contrast and brightness. For the calibration process, 10 CBCT were selected, analyzed, and classified by both operators separately in two times. Using the Cohen’s kappa test, the inter-operator agreement was determined. The result of the calibration indicated an optimal level of inter-operator agreement, as shown by a kappa value of 0.87.

The sample was obtained from the Oral and Maxillofacial Imaging Center of Andres Bello University, Viña del Mar, Chile. CBCT scans performed between 2018 and 2020 were selected using a non-probabilistic sampling for convenience approach until the desired sample size was reached. Scans with bilateral presence of upper first and second molars that presented at least one molar with endodontic treatment of individuals over 18 years of age were included. Exclusion criteria were upper molars with signs of root resorption, previous endodontic surgery, radicular perforation, and any artifact that difficulted the analysis of the area of interest.

The presence of endodontically treated molars was determined using the panoramic view. To evaluate the presence of MB2 missed canals and AP, 0.5 mm slices in the vestibular-palatal direction were used. In cases where visualization was hindered due to the lack of coincidence between the slices and the longitudinal axis of the root, multiplanar reconstruction were used. These were examined according to the longitudinal axis of the root in slices of 0.2 mm every 0.2 mm.

A missed canal was defined as a canal with no evidence of filling material or with filling material exclusively in the cervical third (3). The presence of AP was considered when the continuity of the alveolar cortical bone was interrupted and associated with a hypodense area around the apex or root surface, being at least twice as thick as the normal periodontal ligament space (22).

-Data analysis 

The data obtained were tabulated and analyzed using Stata 11.2 statistical software (StataCorp LLC, USA). The association of MB2 missed canals with AP was calculated using the Fisher exact test. *P* values < 0.05 were considered statistically significant.

## Results

A total of 179 endodontically treated maxillary molars (128 first and 51 second molars) were included for evaluation after analyzing 150 CBCT scans obtained between 2018-2020. Of these, 84 (45.78%) presented untreated MB2 canals, showing a slightly higher frequency for first molars (48.43%) compared to second molars (43.13%).

Sixty-six of the 179 upper molars presented AP (36.8%) and in 46 (70%) of those, the MB2 canal was missed. In the other 20 molars with apical periodontitis, no missed canals were found. The association between missed MB2 canal and apical periodontitis was statistically significant (*p* < 0.0001).

Of the 62 first molars that presented MB2 missed canals, 34 (54.8%) also presented AP, while 28 (45.2%) presented healthy peri-radicular tissues. Twelve first molars without missed canals had also AP. The presence of non-treated MB2 canals was significantly associated with the presence of AP (*p* < 0.0001). Regarding the second molars, 12 (54.5%) presented AP with MB2 missed canals, 10 (45.5%) presented healthy peri-radicular tissues with MB2 missed canal, and 8 presented AP without any missed canal. The presence of non-treated MB2 canals was not associated with the presence of AP (*p* = 0.081).

## Discussion

The location and treatment of MB2 canals in maxillary molars is a daily challenge for clinicians; the access is usually narrow, curved, and frequently covered by secondary dentin (17). These factors explain partly the high percentage of missed canals reported in the literature. Wolcott *et al*., and Yoshioka *et al*., suggested that MB2 missed canals in upper molars may be the main cause of poor long-term endodontic prognosis (22,23).

In this study, we found that 46% of all endodontically treated upper molars presented with MB2 missed canals. This value is slightly higher than the ones reported by others, which is of 40% (3,24). When analyzing upper molars independently, we observed 48.4% and 43.1% of the first and second molars with MB2 missed canals. The higher frequency of MB2 missed canal of the first over the second molar contrasts with the findings reported by do Carmo *et al*., who reported higher frequency of MB2 missed canals in second molars (13). This difference can be attributed to sample distribution, as in our study the number of first molars was considerably higher than second molars.

Post-treatment apical periodontitis is a disease related to endodontically treated teeth affected by persistent or secondary infection (2) and its most frequent causes are related to persistence of bacteria (intra-canal and extra-canal), inadequate root canal filling, overextensions of filling materials, and untreated canals, among others (5). We found that in 70% of all upper molars with apical periodontitis, the MB2 canal was not treated, association that was statistically significant. These results are similar to those reported by Karabucak *et al*., who also found this significant association and concluded that a tooth with a missing canal is 4.8 times more likely to be associated with an apical lesion (3). When analyzing the correlation between AP and MB2 missed canals of the molars separately, we found a statistically significant association for the first molar, but not for the second, which is probably due to the relatively low number of endodontically treated second upper molars included in this study. There were 38 molars (between first and seconds), where the MB2 canal was not treated, but they presented with no apical lesion. This may be associated with anatomical factors, such as high confluence rates between MB1 and MB2 canals (14,25), especially in the South American population (26). The initial diagnosis of the endodontic treatment is also a factor that might explain this. Teeth treated initially for pulpal necrosis that present an untreated canal have a greater chance of causing or maintaining AP than teeth that also have a missed canal but where the pulp was initially vital (or partially vital) (2). Another aspect to consider is the time elapsed between the endodontic treatment and the date of the CBCT scan, as this may influence the results, considering that the time for development and the subsequent imaging visualization of signs of apical periodontitis are variable (27). These are important points to consider, given that in this retrospective study, the sample was randomly drawn from a database without considering symptoms, initial diagnosis, and the indication of CBCT. However, it is important to consider that there are no clinical signs or symptoms that are significant predictors of apical periodontitis visible on CBCT (28).

More than 50% of the first and the second molars where MB2 missed canals were identified, presented also with apical periodontitis. These results are consistent with those reported in the literature, which show a direct relationship between both conditions without differences between first and second molars (2–4,13,24). This highlights the importance of using a clinical dental microscope associated with the selective removal of dentin with ultrasonic tips, which have been described as the most efficient tools for locating MB2 canals (15,23,29) .

## Conclusions

MB2 missed canals are associated to a high degree with apical periodontitis and are an important predictor of endodontic prognosis of upper molars. These results should prompt clinicians to carry out careful treatment planning to maximize the success of endodontic prognosis.
